# High dose CD11c-driven IL15 is sufficient to drive NK cell maturation and anti-tumor activity in a trans-presentation independent manner

**DOI:** 10.1038/srep19699

**Published:** 2016-01-29

**Authors:** Julia K. Polansky, Rajia Bahri, Mylene Divivier, Erwin H. Duitman, Christina Vock, Diego A. Goyeneche-Patino, Zane Orinska, Silvia Bulfone-Paus

**Affiliations:** 1 Research Center Borstel, 23845 Borstel, Germany; 2 Institute of Inflammation and Repair & MCCIR, University of Manchester, Manchester M13 9PT, UK; 3 Facultad de Medicina Veterinaria y Zootecnia, Universidad Cooperativa de Colombia, Bucaramanga, Colombia

## Abstract

The common gamma (γc)-chain cytokine interleukin 15 (IL15) is a multifunctional immune-modulator which impacts the generation, maturation and activity of many cell types of the innate, as well as the adaptive immune system, including natural killer (NK) and CD8^+^ T cells. Using a new series of transgenic mice, we analyzed the *in vivo* potential of IL15 as an immune-regulator when available at different concentrations or delivery modes, i.e. soluble monomer or complexed to its specific receptor α (Rα)-chain. We have identified distinct effects on selected IL15-responsive populations. While CD8^+^ T cells required complexed forms of IL15/IL15Rα for full functionality, mature NK populations were rescued in an IL15/IL15Rα-deficient environment by high levels of CD11c-restricted IL15. These IL15-conditions were sufficient to limit tumor formation in a lung metastasis model indicating that the NK cell populations were fully functional. These data underline the potential of “free” IL15 in the absence of Rα-complex as a powerful and specific immuno-modulator, which may be beneficial where selective immune-activation is desired.

Following its discovery, the cytokine interleukin 15 (IL15) has garnered attention in the basic as well as applied biomedical research fields as an immuno-modulator capable of strongly influencing both, the homeostasis and activation processes of the innate and the adaptive immune system. The essential regulatory role of IL15 in the immune system is clearly demonstrated in IL15-knock-out (*Il15*^−/−^) mice^1^ in which there are reduced numbers and maturation of several leukocyte populations including natural killer (NK), CD8^+^ T, invariant NK-T (iNK-T) cells and intra-epithelial lymphocytes (IELs) in the intestine. In addition, IL15 is required for the activation of NK^2^ and iNK-T cells^3^ and enhances the cytotoxic capacity of NK cells^4^.

The heterotrimeric receptor of IL15 is composed of the private IL15Rα-chain, the IL2R/IL15Rβ-chain (CD122, also part of the IL2 receptor complex) and the common gamma (γc)-chain^5^,^6^, the latter being part of receptor complexes for a number of cytokines. The transcription, splicing, and translation of IL15 are tightly controlled through a multi-layered regulatory process^7^,^8^. Furthermore, the intracellular shuttling and secretion of IL15 have been shown to rely on IL15Rα expression^9^,^10^,^11^.

In contrast with many other cytokines, IL15 has been reported to mediate its physiological activity not as a secreted monomer, but predominantly complexed with the high affinity IL15Rα^6^. The IL15/IL15Rα-complex is localized to the plasma membrane of IL15-producing cells and presented to target cells bearing the IL15Rβ and γc-chains in a process termed ‘trans-presentation’. Owing to its extraordinary signaling mechanism, transmission of IL15 signals via this route requires cell-cell contact and is thus restricted to neighboring cells^12^. This trans-presentation mechanism and the need for cell-cell contact highlights that the cellular source of IL15 is critical to determining the effects of IL15. However, following trans-presentation, IL15/IL15Rα-complexes may be shed from the plasma membrane^13^, and enter the bloodstream where they can be detected in serum samples^11^. Furthermore, it has also been shown that IL15 complex formation with IL15Rα prolongs the bioavailability of recombinant IL15^14^.

While physiological IL15 is a critical component of a protective immune system, its elevated expression has been observed in multiple autoimmune diseases, such as rheumatoid arthritis^15^, multiple-sclerosis^16^ and systemic lupus erythematosus^17^, with studies highlighting IL15 as a potential therapeutic target. Conversely, harnessing the powerful immunostimulatory effects of IL15 has shown potential as a cancer immunotherapy agent and clinical phase I trials are currently underway (reviewed in^18^). Under physiologic conditions the activities of IL15 are tightly regulated, however it is evident that in pathologic settings dysregulated IL15 may be either disadvantageous as is the case in autoimmunity, or beneficial in boosting the immune response to cancer. It is therefore of the utmost importance to delineate the complex multifaceted roles of IL15 and to determine IL15-mediated effects *in vivo* under well-defined conditions.

In the present study, we analyzed the *in vivo* effects of ‘free’ IL15 or IL15/IL15Rα complexes using a series of newly generated transgenic mice. These mice express IL15 under the control of the CD11c minimal promoter, which largely restricts IL15 expression to dendritic cells (DCs), which are one of the main, although not only, IL15-expressing cell type in wildtype mice.

To our surprise, we found distinct requirements for different lymphocyte populations concerning both, the mode of IL15 delivery and the required IL15 expression levels. Most interestingly, mature NK cells, but not CD8^+^ T cells, could be reconstituted in IL15-deficient (*Il15*^−/−^) mice by providing high levels of CD11c-restricted ‘free’ IL15 only. These cells appear to be fully functional as tumor growth in a lung metastasis model was largely inhibited. With this we show, that, in contrast to the current consensus in the field, IL15 harbors the potential to recover functional NK populations even in the absence of IL15Rα when expressed in a cell type-restricted manner.

## Results

### Generation of IL15-transgenic mice expressing different levels of IL15 under the control of the CD11c promoter

To limit the spectrum of IL15-producing cells and in order to evaluate the role of cell type-restricted IL15 expression levels on lymphocyte differentiation and homeostasis, we generated four mouse strains in which the *Il15* gene was expressed under the control of the CD11c promoter. By crossing these novel strains onto the *Il15*^−/−^ background, we limited IL15 expression in these mice to CD11c expressing cells.

**Figure 1:**
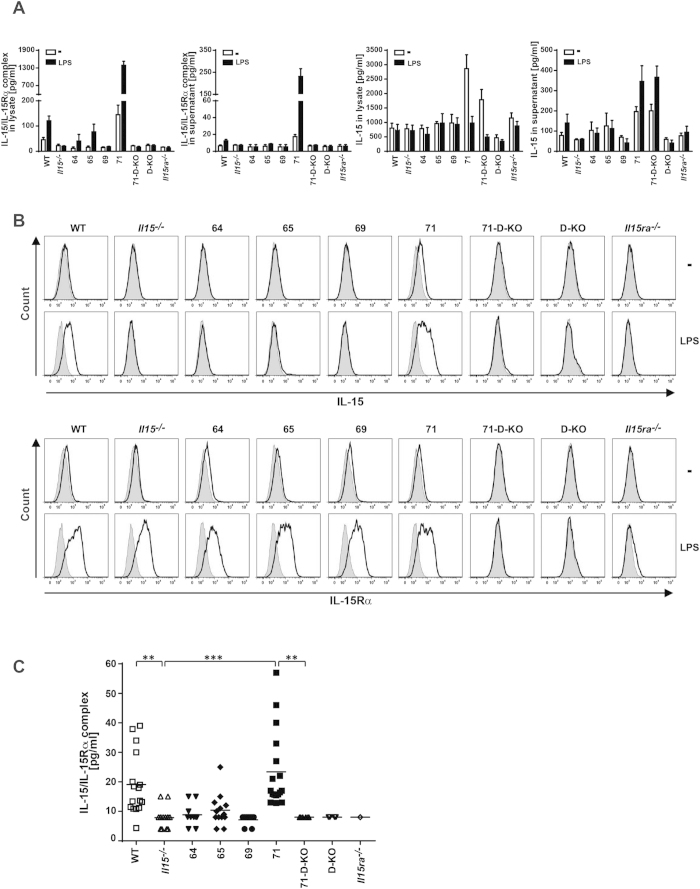
The IL15-transgenic strains express different levels of IL15. **(A)** *In vitro* generated BMDCs were treated with LPS for 24 h or left untreated (−) and IL15 and IL15/IL15Rα complexes were quantified by ELISA in the cell lysates and supernatants (n = 3–8). **(B)** Surface BMDC IL-15 and IL-15Rα expression was measured by flow cytometry. Grey filled histograms represent the isotype control, black lines show IL15 or IL15Rα staining. Representative staining of 3 independent experiments is shown. **(C)** IL15/IL15Rα complex levels were quantified by ELISA in the sera of the different mouse strains. Statistics: ***p<=0.001; **p<=0.01.

We analyzed IL15 expression patterns in all four *CD11c-Il15* strains (indicated as 64, 65, 69 and 71) and observed comparable numbers of CD11c^+^ cells in the spleen (Supplementary Fig. S1A), but distinct expression levels of transgenic IL15 between the strains. Cell lysates from CD11c^+^ bone marrow-derived dendritic cells (BMDCs) were analyzed using two different ELISAs, one detecting IL15/IL15Rα-complexes and one detecting uncomplexed (“free”) IL15 (Fig. 1A). High levels of free IL15 were detected in BMDC lysates of strain 71 with some release of free IL15 into the cell culture supernatant. There were no detectable levels of free IL15 in BMDC lysates derived from transgenic mouse strains 64, 65 and 69, with levels comparable to that of values obtained from *Il15*^−/−^ BMDC lysates, used here as a negative control for the assay. This control was essential as the ELISA for IL15 appears to be more sensitive in its detection of recombinant IL15 compared with naturally occurring IL15, as can be seen by the high background levels in our BMDC lysates (Fig. 1A). Only low levels of IL15/IL15Rα complexes could be detected in BMDC lysates from strain 71 under steady state conditions, indicating that most of the overexpressed transgenic IL15 was present in the unbound form, probably due to low expression of IL15Rα as demonstrated by IL15Rα surface staining (Fig. 1B). However, stimulation of BMDCs with lipopolysaccharide (LPS) resulted in uniformly present IL15Rα surface expression in all BMDC genotypes (Fig. 1B) and increased levels of IL15/IL15Rα complexes in lysates of C57BL/6 wildtype (WT), 64, 65 and 71 mice (Fig. 1A) with shedding of IL15/IL15Rα complexes from the surface into the culture supernatant at low levels from WT and substantially higher levels from strain 71 (Fig. 1A). In WT and strain 71 mice, IL15 could be detected on the surface of BMDCs following LPS-stimulation (Fig. 1B), confirming IL15/IL15Rα complex formation and surface trans-presentation. Interestingly, lysate concentrations of free IL15 following LPS stimulation were comparable to background values in all samples (Fig. 1A) indicating full complex formation and/or release into the supernatant in response to stimulation.

*In vivo*, systemic levels of the IL15/IL15Rα complex in serum were reconstituted in the 71 mice to levels comparable with WT mice values (Fig. 1C). This result demonstrates that CD11c^+^ restricted overexpression of IL15 is sufficient to fully reconstitute systemic circulating IL15/IL15Rα complex levels. Free IL15 in the serum could not be detected in any strain (data not shown), indicating that free IL15 may only be present in the vicinity of CD11c^+^ producing cells, exerting its influence locally, rather than disseminating and having a systemic effect. However, due to the low sensitivity of the IL15 ELISA we cannot exclude the presence of low levels of free IL15 in the serum.

Taken together, these data allow the 6 analysed mouse strains to be ranked according to their relative levels of IL15 expression in comparison to *Il15*^−/−^ =/< 69 < 64 < 65 < WT < 71. Furthermore, CD11c^+^ DC maturation/activation status was found to be comparable in in all mouse strains analysed, indicating a redundant role for IL15 in the generation and maintenance of these cells (Supplementary Fig. S1B).

### Genetic IL15Rα-deletion facilitates overexpression of free IL15 in the transgenic mouse strain 71

To determine the specific roles of CD11c-produced free IL15 *versus* complexed IL15, we bred mouse line 71 on an *Il15R*α^−/−^*/Il15*^−/−^ (=double knock-out, D-KO) background. As expected, no IL15/IL15Rα-complexes could be detected in BMDCs generated from this strain (71-D-KO), however there were high levels of free IL15 in both the lysates and cell culture supernatants, with secretion increased upon LPS stimulation (Fig. 1A). Furthermore, neither membrane-bound IL15 on BMDCs (Fig. 1B) nor serum IL15/IL15Rα-complexes (Fig. 1C) could be detected. Thus, the newly generated mouse strains 71 and 71-D-KO are suitable to investigate the role of trans-presented *versus* soluble IL15 by CD11c^+^ cells, respectively.

### CD8^+^ T cells are gradually reconstituted with increasing levels of CD11c-restricted trans-presented but not free IL15

**Figure 2:**
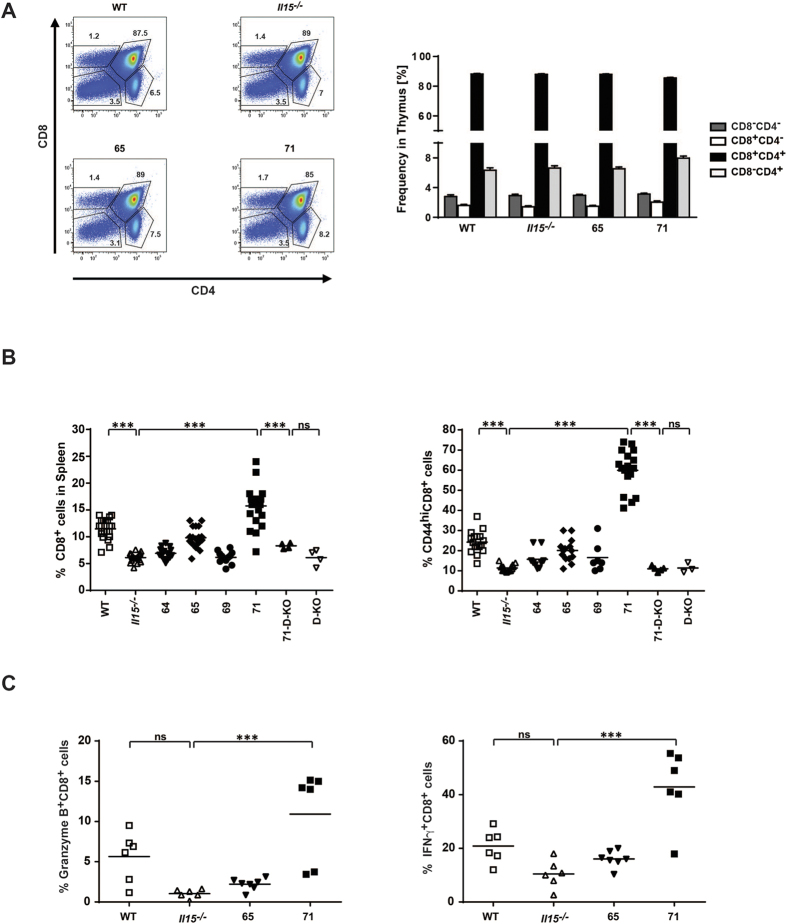
The peripheral CD8^+^ T cell population is re-established with high levels of trans-presented IL15. **(A)** Thymocyte surface expression of CD8 and CD4 analysed by flow cytometry (left), bar diagram quantifies percentages of cells in different T cell maturation stages (right, n = 6–7). **(B)** Percentage of CD8^+^ (left) and CD44^hi^CD8^+^ T cells (right) in the spleen as quantified by flow cytometry. **(C)** Splenocytes were isolated and stimulated with IL2 (left) or with agonistic anti-CD3 and anti-CD28 antibodies (right), 24 hours later cells were stained for surface CD8 and intracellular GzB (left) or intracellular IFNγ (right) and analysed by flow cytometry. Statistics: ***p<=0.001; ns = non significant.

IL15 is required for the homeostasis and development of memory CD8^+^ T cells. Therefore we examined CD8^+^ T cell populations in the spleen and the thymus of all generated transgenic mouse strains. As expected, none of the IL15-transgenic strains displayed abnormal thymic T cell development (Fig. 2A). However, in the spleen, both, the frequency (Fig. 2B) and total number (data not shown) of CD8^+^ T cells were found to gradually (although not statistically significantly) increase with increasing amounts of trans-presented IL15 (*Il15*^−/−^ < 65 < WT < 71). In line with the known dominant role of IL15 on the expansion of memory CD8^+^ T cells^19^, the majority of CD8^+^ T cells in the IL15 overexpressing mouse strain 71, displayed a memory phenotype (CD44hi, Fig. 2B), which correlated with increased expression of the effector molecules granzyme B (GzB) and interferon gamma (IFNγ) (Fig. 2C).

Most strikingly, the 71-D-KO strain did not show significant CD8^+^ T cell reconstitution in the spleen (Fig. 2B) despite the strong overexpression of free IL15 in these mice, as well as a comparable IL15 transcript levels between 71 and 71-D-KO mice and at levels higher than WT mice (Supplementary Fig. S2A), indicating that CD8^+^ T cells are strictly dependent on IL15 trans-presentation.

### Intra-epithelial T cells and thymic NK-T cells are not reconstituted by CD11c-restricted IL15

**Figure 3:**
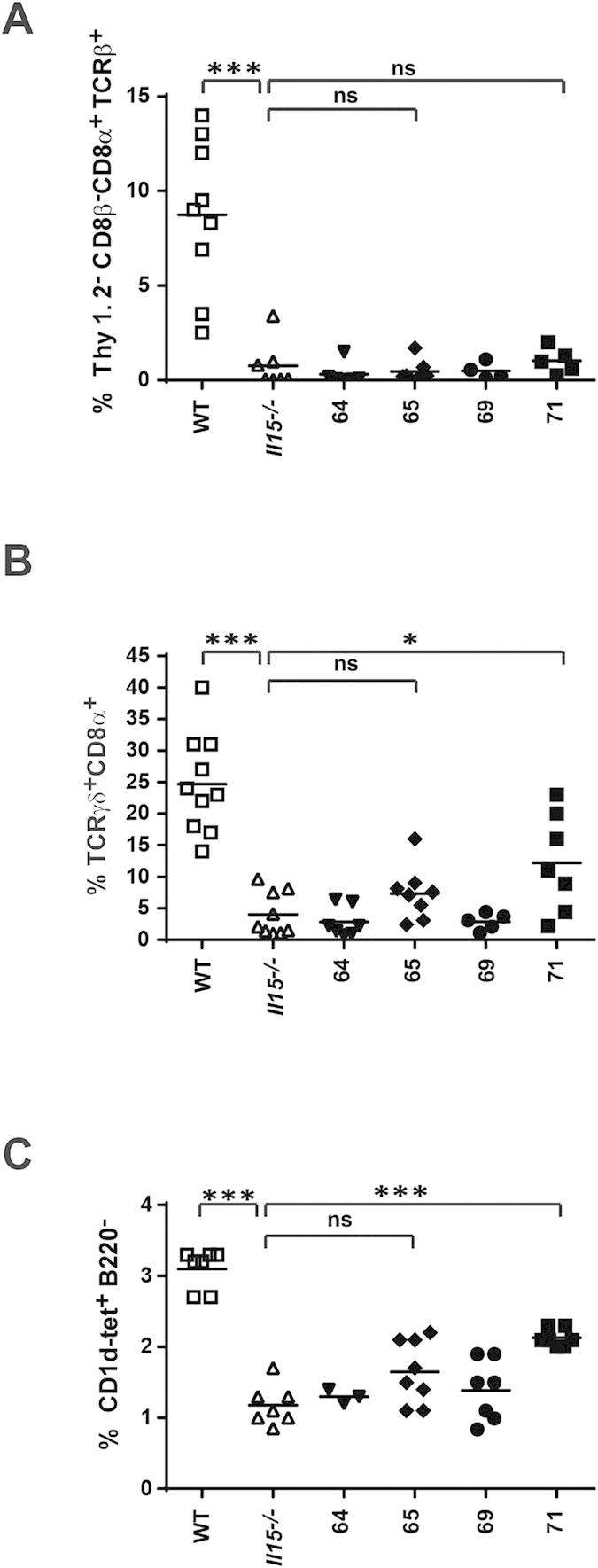
IELs and iNK-T cells cannot be fully re-constituted with CD11c-derived IL15. CD8αα **(A)** and TCRγδ^+^ **(B)** IELs were isolated from the small intestine and identified by flow cytometry using the surface marker combinations indicated. iNK-T cells **(C)** were identified by staining with the PBS-57 CD1d-tetramer (CD1d-tet) from thymocytes. Statistics: ***p<=0.001; *p<=0.05; ns = non significant.

In contrast to circulating CD8^+^ T cells, which are dependent on trans-presented IL15-levels rather than free IL15, and hence, may be rescued by CD11c-restricted IL15 overexpression, other lymphocyte populations appear to have more stringent requirements. Intra-epithelial T lymphocytes (IELs) in the gut have been described to rely on trans-presentation of IL15 provided by neighboring gut-epithelial cells^20^,^21^. Our own results strongly support this finding, as both types of IELs (TCRαβ^+^ CD8αα^+^ Thy1^−^ or TCRγδ^+^ CD8α^+^) are not restored in our CD11c-restricted IL15-expressing mice, even in strain 71 where IL15 is highly overexpressed (Fig. 3A,B). A similar scenario seems to account for the development of invariant NK-T (iNK-T) cells in the thymus, which largely rely on the trans-presentation of IL15 by thymic parenchymal cells (Fig. 3C)^22^,^23^. Despite the presence of CD11c^+^ DCs in the thymus, they are unable to restore the complete iNK-T cell compartment, even in the presence of abundant trans-presented IL15.

### The size of the NK-population is reconstituted in different organs with increasing levels of trans-presented but not free CD11c-restricted IL15

**Figure 4:**
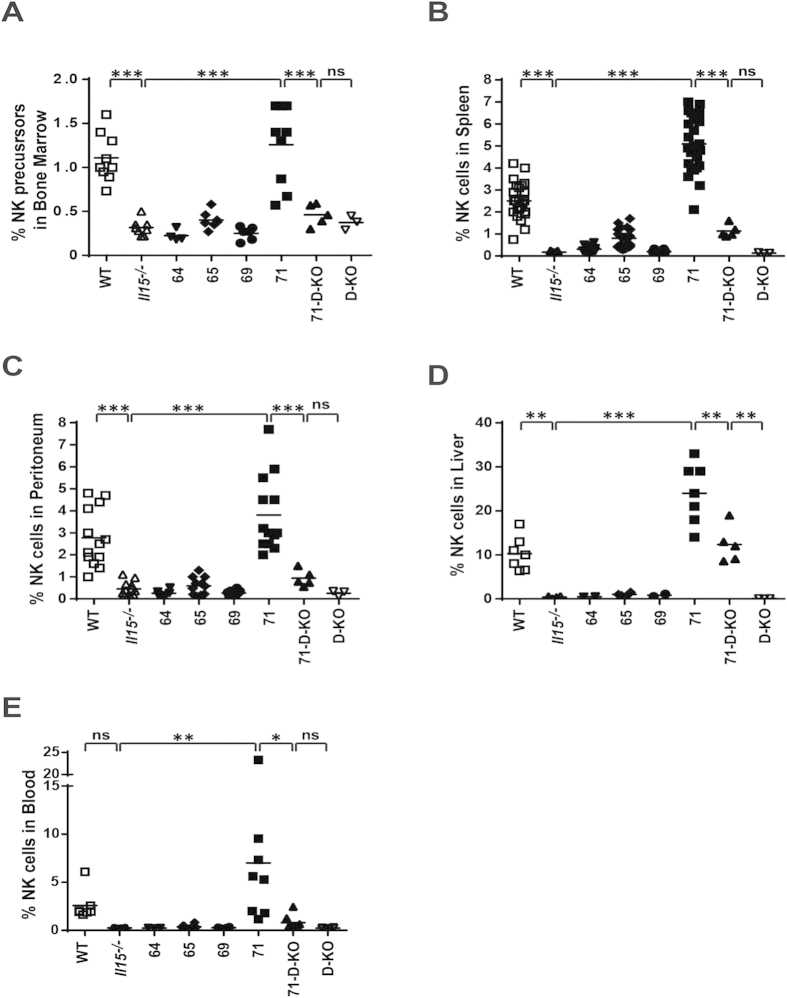
The NK cell population is re-established with high levels of trans-presented IL15. Frequencies of NK precursor cells (CD122^+^NK1.1^−^) were analyzed in the bone marrow **(A)** while frequencies of NK cells were analysed in spleen **(B)**, peritoneum **(C)**, liver **(D)** and blood **(E)** by flow cytometry. Statistics: ***p<=0.001; **p<=0.01; *p<=0.05; ns = non significant.

NK cells are known to be dependent on IL15 for their maturation and survival. Frequencies of NK cell precursors (CD122^+^NK1.1^−^)^24^ in the bone-marrow were reconstituted in line with WT levels in strain 71 and achieving partial reconstitution in strain 65 (Fig. 4A). Furthermore, we found the percentage of NK cells in both blood and peripheral tissues (spleen, peritoneum and liver) to be fully reconstituted to WT levels (or above) in strain 71 (Fig. 4B–E). We also observed an intermediate, although non-statistically significant, reconstitution in strain 65 (Fig. 4B,C). Upon genetic deletion of the IL15Rα-chain (71-D-KO), reconstitution of NK cells was comparable with WT levels in the liver (Fig. 4D), however, only partial reconstitution was achieved in the bone marrow, spleen, peritoneum and blood (Fig. 4A–C,E) despite high levels of free IL15 (Fig. 1A). These results indicate that the successful generation and survival of NK cells is determined by levels of trans-presented IL15 (mouse strains 71 vs. 65), while the ability of free IL15 to influence this process is dependent on the target organ (e.g. liver vs. spleen).

### NK cell maturation in the bone-marrow and in the spleen is independent of IL15 trans-presentation

**Figure 5:**
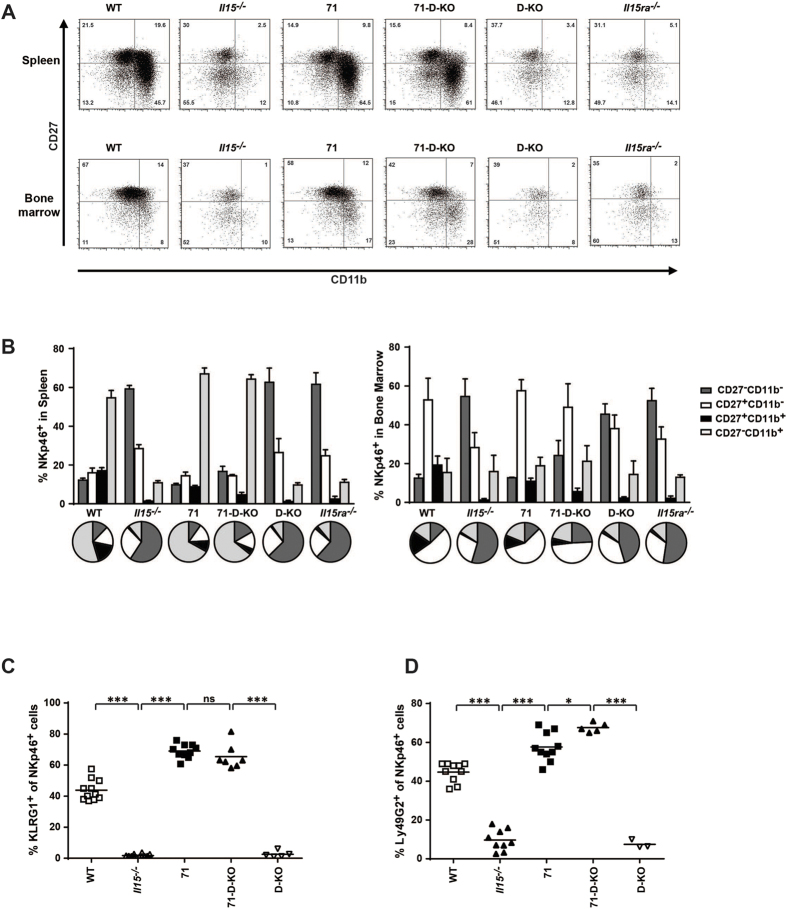
IL15-driven maturation of NK cells occurs independently of IL15 trans-presentation. The maturation state of NKp46^+^ NK cells was analyzed flow cytometry. **(A)** Representative FACS plots of CD27 and CD11b staining on NKp46^+^ cells in the spleen and bone marrow. **(B)** Summarized frequencies of NK cell maturation stages in spleen and bone marrow (n = 4–7). Percentages of **(C)** KLRG1^+^ and **(D)** Ly49G2^+^ in NKp46^+^ cells in the spleen. Statistics: ***p<=0.001; *p<=0.05; ns = non significant.

NK cell maturation is typically defined by the expression of CD27 and CD11b (Mac-1), with four distinct stages (least mature = CD27^−^ CD11b^−^ -> CD27^+^ CD11b^−^ -> CD27^+^ CD11b^+^ -> CD27^−^ CD11b^+^ = most mature), with maturity correlating with the progressive acquisition of NK cell effector functions^25^. Increased maturity of the CD27^−^ CD11b^+^ NK cell stage can be further characterized by high expression of the inhibitory lectin-like receptor KLRG1^26^. NK cells surviving in an IL15-deficient environment show an immature phenotype, with low CD11b and KLRG1-expression, thus indicating that IL15 is not only required for the generation and survival of NK cells, but also for their full maturation (Fig. 5A–C). In accordance with these observations, NK cells from the CD11c-driven IL15 overexpressing mice, strain 71, display a fully mature phenotype in the spleen with the majority of the NK cells being CD27^−^ CD11b^+^. Furthermore, the majority of NKp46^+^ NK cells in strain 71 are KLRG1^+^, further indicating the fully mature NK phenotype in this strain and the importance of IL15 to NK cell maturation (Fig. 5A–C). Interestingly, in strain 71-D-KO, in which IL15 trans-presentation is abolished, a similar degree of maturation to strain 71 was observed in the NK population, suggesting that unlike generation/survival of NK cells, their maturation is independent of IL15 trans-presentation and may be rescued by high levels of free IL15 (Fig. 5A–C). This rationale also applies to the maturation stages in the bone marrow, in which developing NK cells in strains 71 and 71-D-KO showed a similar CD27/CD11b profile as WT mice and were markedly distinct from cells in *Il15*^−/−^, *Il15R*α^−/−^ and D-KO mice (Fig. 5A,B). Similarly, the inhibitory NK surface receptor Ly49G2 was expressed by splenic NK cells from WT, 71 and 71-D-KO mice but not from *Il15*^−/−^ and D-KO mice (Fig. 5D), further confirming the full maturation of NK cells in the absence of IL15 trans-presentation.

**Figure 6:**
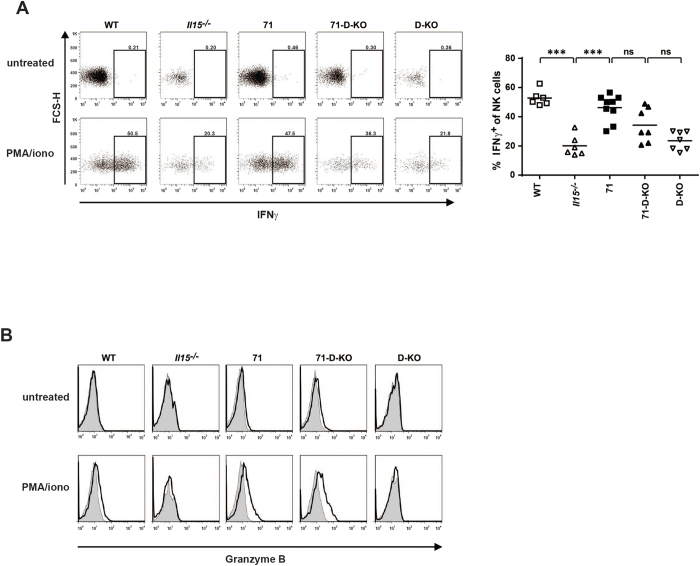
NK cells from IL15 trans-presentation deficient mice are fully functional *in vitro.* Splenocytes were stimulated with PMA/ionomycin or not and stained for **(A)** intracellular IFNγ or **(B)** GzB. Grey filled histograms show the isotype control, black lines represent the GzB staining. Plots were gated on NK cells and show one representative example in (**A)** left panel (n = 6–9) and (**B)** (n = 2). (**A)** right panel shows summarized percentages of IFNγ^+^ NK cells. Statistics: ***p<=0.001; ns = non significant.

To confirm the functional competence of mature NK phenotypes, we analyzed GzB and IFNγ expression in splenic NK cells after stimulation *in vitro* using intracellular staining and flow-cytometry. In accordance with their phenotypically mature state, we found significant IFNγ production (Fig. 6A) and increased GzB expression (Fig. 6B) in response to PMA/Ionomycin in NK cells from mouse strains 71 and 71-D-KO while cells from *Il15*^−/−^ mice showed reduced effector functions in line with their phenotypically immature status.

Taken together, our data suggest, that some of the IL15-mediated effects (NK maturation and functionality), which were traditionally thought to be trans-presentation dependent, are mediated by soluble IL15 if provided at high enough levels. However, other IL15 mediated processes remain strictly dependent on trans-presentation of IL15 (e.g. CD8^+^ memory T cell survival) or on certain cell-types as sources of IL15 (e.g. IEL maintenance).

### Metastatic colony formation is impaired by CD11c-restricted IL15 overexpression independently of trans-presentation

**Figure 7:**
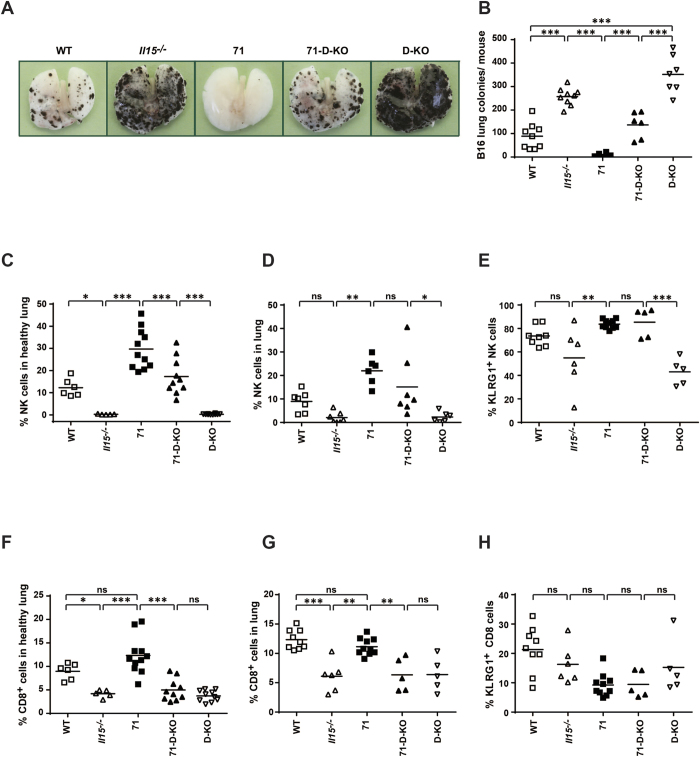
Lung metastasis is impaired by CD11c-restricted IL15 overexpression independently of trans-presentation. Recipient mice of the genotype indicated were injected *i.v.* with 2 × 10^e^5 B16 melanoma cells. Macroscopic lung metastases **(A)** were counted **(B)**. Lung cells were isolated and analysed by flow cytometry for frequencies of **(D)** NK cells, **(E)** KLRG1^+^ NK cells, **(G)** total CD8^+^ T cells and **(H)** KLRG1^+^ CD8^+^ T cells. Frequency in healthy lungs of total **(C)** NK cells and **(F)** CD8^+^ T cells are also shown. Statistics: ***p<=0.001; **p<=0.01; *p<=0.05; ns = non significant.

NK cells have previously been shown to play a major role in impairing the development of lung metastases^27^. Therefore, we investigated the ability of both free and complexed CD11c-restricted IL15 to inhibit lung metastasis in a model of metastatic melanoma. B16 melanoma cells were injected intravenously and metastatic colonies in the lungs were counted on day 19 post injection. Lung metastases were dramatically increased in the absence of IL15 (*Il15*^−/−^ and D-KO) compared with WT (Fig. 7A,B). However, overexpression of CD11c-derived IL15 completely protected strain 71 from metastasis (Fig. 7A,B). Interestingly, the 71-D-KO mouse strain also showed only limited numbers of metastatic foci in the lungs compared to D-KO mice, indicating that the protective effect of transgenic IL15 in these mice is trans-presentation independent and, hence, relies on the presence of mature NK cells. Indeed, in this metastasis model NK cells were strongly increased in the lungs of strain 71 and partially increased in strain 71-D-KO (Fig. 7D) compared to their controls (*Il15*^−/−^ and D-KO, respectively), along with increased frequencies of KLRG1^+^ mature NK cells (Fig. 7E). The numbers of tumor foci inversely correlated with KLRG1^+^ NK cell frequencies, therefore indicating an important role of these cells in controlling metastasis formation. High frequencies of NK cells were also detected in the healthy lungs of untreated 71 and 71-D-KO mice (Fig. 7C), indicating that a proportion of the protective NK cells are potentially lung-resident, rather than recruited. This assumption is supported by an equally high expression of IL15 in healthy lungs of both the 71 and 71-D-KO mice (Supplementary Fig. S2B). In contrast, CD8^+^ T cells were comparable in frequency between WT and strain 71 in both healthy and lungs of tumor-bearing recipients (Fig. 7F,G), with only a small proportion of CD8^+^ T cells in all mouse strains being KLRG1^+^ after metastasis formation (Fig. 7H), indicating that in this model CD8^+^ T cells do not play a major role inhibiting metastasis.

## Discussion

In this study, we have investigated the *in vivo* actions of IL15 firstly as a soluble mediator and secondly in complex with IL15Rα. We suggest that while CD8^+^ T cells require complexed forms of IL15/IL15Rα for full functionality, mature NK populations rely on IL15 but not IL15Rα expression. Thus, arguing that free IL15 alone is not only sufficient in anti-tumor therapies, but could potentially be better tolerated as a therapeutic by predominantly targeting NK cells and avoiding overwhelming CD8^+^ T cell activity.

In our study, we analyzed the influence of IL15 on the *in vivo* development and activity of NK and CD8^+^ T cells in situations of restricted IL15 expression with respect to the 1) levels of expression (low to high), 2) the cellular source (CD11c^+^ cells, mainly dendritic cells (DCs), and 3) the mode of action (IL15Rα-complexed or monomeric) utilizing a series of new IL15 transgenic mouse strains. Of note, this genetic approach results in constitutive (over) expression of IL15 in CD11c^+^ cells, a gene, which, in DCs, is usually regulated in response to activation. In these mice, we are therefore *not* assessing physiological conditions, but instead assessing the functions of CD11c^+^-derived IL15 at different expression levels and through different delivery modes (free *versus* equal levels of trans-presented IL15). Expressing IL15 under the control of the CD11c promoter ensures its expression in cell types, which physiologically do express the cytokine in a tightly regulated manner and also ensures the expression of the cytokine at organs and sites in which tumor immunity is occurring.

Under these genetic conditions we found several interesting results concerning the mode of IL15 delivery. Firstly, we confirm earlier published studies reporting that increasing levels of IL15 trans-presentation gradually restore the NK cell compartment^28^ as well as CD8^+^ T cell numbers. Secondly, we show that high levels of IL15 are able to overcome the reported dependency of NK^20^,^29^ and CD8^+^ T cells^20^,^30^ on diverse cellular sources of IL15, while IELs^20^,^21^ and iNK-T^22^,^23^ remain largely dependent on particular (most likely neighboring) cells as IL15 trans-presenters. Thirdly, we found that ‘free’ (uncomplexed) IL15 showed an unexpected capacity for the maturation of NK cells when expressed at high levels. Interestingly, high levels of free IL15 correlated with an efficient increase in NK cells in non-lymphoid organs (lung and liver), in which the frequency of NK cells in the lymphocyte population has been found to be the highest^31^, indicating an increased sensitivity towards free IL-15 in non-lymphoid organs for the maintenance/generation of NK cells. Under these special conditions, metastasis development in the lung was strongly inhibited, indicating that these free IL15 generated NK cells were fully functional. This conclusion is in line with literature reporting prevention of lung or breast cancer metastasis being dependent on mature KLRG1^+^ NK cell numbers^27^,^32^ and IFNγ-mediated mechanisms^33^. Furthermore, our findings on the role of IL15-driven NK cell-mediated inhibition of tumor growth are in accordance with our previous report using the DSS/AOM model of colitis-associated colon carcinogenesis^34^ and the findings that IL15 induces very densely granulated NK cells that can eliminate large established solid tumors in the absence of T and B cells^35^. In this latter model expression of the IL15Rα on cancer cells was needed to efficiently induce granulated NK cells, and expression on host stromal cells was essential to prevent tumor relapse. Furthermore, the expression of IL15 in the tumor microenvironment by cancer cells has been described to potentiate antigen-independent T cell cytotoxicity and tumor eradication^36^. Interestingly, in the lungs of healthy untreated 71 and 71-D-KO mice we observed high frequencies of NK cells independently of IL15Rα expression thus indicating that not only IL15 concentration but also the cellular source could be essential in maintaining NK cell functionality. In our study it remains unclear, and therefore requires further investigation, whether the expression of IL15 and/or IL15Rα at the tumor site has any relevant/conditional effect in tumor growth inhibition.

In our CD11c-driven model, we cannot fully exclude that some IL15 production in the 71 and 71-D-KO mouse strains could originate from cells other than antigen presenting cells such as activated CD8^+^ T and NK cell subsets^37^. Despite the fact that this expression could lead to an autocrine positive feedback loop in mouse strain 71, the differences observed in the 71-D-KO strain are striking. Thus, in the latter strain, NK cell maturation remains IL15 trans-presentation independent.

Taken together, we have come to the conclusion that IL15/IL15Rα complex formation is not a strict requirement for NK cell maturation when free IL15 concentrations reach a threshold that guarantees NK cell functionality even when the cellular source of IL15 is restricted. Our data are not only in agreement with the results of the first human clinical trial using recombinant human IL15 in patients with cancer^38^ but also provide the basis of a mechanistic explanation of the results and are of importance for future therapeutic approaches in the prevention of metastasis formation. While IL15 has shown therapeutic promise, systemic use of IL-15 in patients or the overexpression of IL15 in mice leads to potentially dangerous side effects. Our data demonstrated the advantage of cell type restricted production/delivery of IL15 in comparison to the intravenous IL15 administration used in human IL15 trials^38^. Cell type restricted expression of IL15 could potentially be harnessed as a therapeutic towards cancer through the *in vitro* expansion of NK cells for immunotherapy and the *in vitro* generation of DCs for induction of tumor-antigen specific CD8^+^ T cell response^39^, in an effort to limit immunotoxicity observed in global IL15 administration. Furthermore, the utilization of IL15 in combination therapy with other cytokines (e.g. IL-21 or GMCSF), or co-stimulatory molecules (CD40) is a potential avenue to be explored to improve current cancer immunotherapy.

In addition, our data point to the necessity of a thorough re/evaluation of the mode-of-action of cytokines and possibly other soluble mediators with respect to their cellular source, dose, their bioactive structure as well as their area of influence. While for most cytokines the bioactive form is believed to be the secreted monomer, which is able to reach even distant locations via the blood stream, this view might be an oversimplification for certain mediators. As an example, it has been described that DCs are able to produce IL2^40^, a cytokine commonly believed to be exclusively expressed by T cells. IL-2 was delivered to neighboring T cells together with the high-affinity IL2Rα-chain (CD25), in a trans-presentation-like process^41^. As IL2-producing DCs did not express the IL2Rβ-chain, they seem to express CD25 exclusively for the purpose of IL2 trans-presentation and the activation of CD25^−^ T cells in their vicinity. These results and our own data underline the need for detailed analyses of the mode-of-action of soluble mediators, especially when these factors are being considered as therapeutic agents (such as IL15 and IL2). Despite the numerous differences between human and murine NK cells, many findings in mouse models which describe the ontogeny, function, and regulation have been confirmed in human studies, therefore suggesting a possible translation of our findings to the benefit of novel clinical therapeutics^42^.

In summary, we suggest that IL-15Rα expression plays a critical role for the development and effector functions of CD8^+^ T cells in IL15-deficient mice in which high levels of CD11c-restricted IL15 is provided. Despite trans-presentation being an important process for adequate CD8^+^ T cell immunity, NK cells were capable of inhibiting tumor development in the absence of IL15Rα exprseesion in our *in vivo* model of tumor metastasis. Sato and colleagues^43^ observed the development of CD8^+^ leukemia founder cells upon the stable expression of IL15 and IL15Rα in a mouse IL15 transgenic model. Therefore, we share their view on the need of caution and propose that the clinical administration of recombinant IL15 alone could be efficient and sufficient in NK cell-mediated tumor eradication thus avoiding potentially immunostimulatory molecules generated by the combination of IL15Rα with IL15.

## Materials and Methods

### Mice

C57BL/6j (WT), *Il15*^−/−^ 1, *Il15r*α^−/−^ 44 and *Il15R*α^−/−^*/Il15*^−/−^ (=double knock-out, D-KO) mice were on the C57BL/6 background and were housed under specific pathogen-free conditions at the Animal Care Facility, Research Center Borstel and used from 8 weeks of age. Animal experiments were performed in accordance with institutional guidelines and were approved by the local authorities (Ministry of Agriculture, the Environment, and Rural Areas, Schleswig-Holstein).

For the generation of transgenic mice expressing CD11c-restricted IL15, a vector containing the CD11c minimal promoter was used as previously described^45^,^46^ and was kindly provided by Tim Sparwasser (Institute of Infection Immunology, Twincore, Medical School Hannover, Germany). The coding sequence of IL15 was amplified by PCR using the following primers: primer 1: 5′-CCCGAATTCCCACCATGAAAATTTGAAACC-3′ and primer 2: 5′-CCCGAATTCTCAGGACGTGTTGATGAACAT-3′. The 0.5 kb fragment was cloned 3′ of the CD11c minimal promoter. After linearization and purification, the construct was injected into C57Bl/6 blastocysts. Positive founder animals were identified by PCR analysis of ear-punch DNA. The resulting offspring were screened for the presence of the transgene by PCR to detect the CD11c promoter/IL15-transgene using the following primers: primer 3: 5′-GGTCTCTGGCCTCCTGAC-3′ and primer 4: 5′-CAGGACGTGTTGATGAACATTTGG-3′. All transgenic strains were heterozygous for the transgene and backcrossed to *Il15*^−/−^ mice.

### Cell Culture

BMDCs were generated from bone marrow cells as described previously^47^. Briefly, bone marrow cell were plated on tissue culture dishes (Sarsted) and cultivated in complete RPMI-1640 medium (Gibco Life Technology) supplemented with 10% FCS (Biochrom), 10 U/ml Penicillin, 0.1 mg/ml Streptomycin, 2 mM L-Glutamine (all from PAA), 0.5 mM 2-βMercaptoethanol (Gibco Life Technology) and 20 ng/ml rmGM-CSF (Biolegend) for 6–7 days. Resulting BMDCs were stimulated with 100 ng/ml LPS derived from *Salmonella enterica* serovar Friedenau^48^ for 24 h, with supernatant collected for further analysis. Cells were detached from plates using Accutase (eBioscience) and washed. To generate cell lysates, 2.5 × 10^e^6 cells were lysed in 250 μl ice-cold lysis buffer (25 mM Tris-HCl, 75 mM NaCl, 1% ODGP, 10 μg/ml Pepstatin A, 10 μg/ml Leupeptin, 1 mM Na-vanadate, pH 7.5) for 15 min on ice then centrifuged at 13000 × g for 10 min. The cleared cell lysates were aliquoted and stored at −80 °C.

For stimulation of NK and CD8^+^ T cells, total splenocytes were harvested and cultured in complete RPMI-1640 cell culture medium, supplemented with 10% FCS, 10 U/ml Penicillin, 0.1 mg/ml Streptomycin, 10 mM HEPES (PAA), 0.5 mM 2-βMercaptoethanol, 1 mM Na-Pyruvate (Gibco Life Technology). CD8^+^ T cells stimulation was achieved using plate-bound anti-CD3 (17A2) and anti-CD28 (37.51; 1 μg/ml each, both eBioscience) or 100U human recombinant IL2 (Biotest Pharma) for 1 day. NK cells were stimulated using phorbol myristate acetate (PMA, 100 ng/ml) and ionomycin (1 μM; both from Sigma) for 6 h. For intracellular cytokine staining, Brefeldin A (10 μg/ml, Sigma) was added to cultures for 4 h to inhibit cytokine release. Following stimulation, cell surface, intracellular and live/dead staining was performed as described below.

### *Ex vivo* cell isolation

Lymphocytes were recovered from the thymus and spleen by straining the organs through an EASYstrainer (70 μm, Greiner-bio-one). Bone marrow cells were flushed with PBS from dissected femurs and tibiae. Peritoneal lavage was performed by injection and subsequent recovery of 5.0 ml sterile 0.89% NaCl from the peritoneal cavity. Blood was diluted with 2.7% EDTA in PBS for leukocyte recovery. Undiluted blood was incubated for 1 h at 37 °C and centrifuged to recover serum. For IELs, the small intestine was removed and the fecal content cleared. After a longitudinal cut, the tissue was washed twice with DMEM medium containing 5% FCS, and then cut into small pieces. IELs were extracted by shaking in freshly made stripping buffer (5% FCS in PBS with 5 mM EDTA and 1 mM DTT) for 30 min at 37 °C. For lung lymphocytes, dissected lungs were cut into small pieces and digested with DNAse (30 μg/ml) and Collagenase (0.7 mg/ml) (both Sigma) for 30 min at 37 °C and 180rpm. Cell suspensions were filtered through an EASYstrainer (100 μm) and washed by centrifugation in HBSS/3%FCS at 420 × g for 5 min at 4 °C. Resulting pellets were diluted in HBSS/3%FCS and lung lymphocytes were obtained by centrifugation using Lympholyte (Cedarlane) according to manufacturer’s protocol. For flow cytometry, erythrocytes were lysed, where required, for 5 min on ice using a lysis buffer (0.15 M NH_4_Cl, 10 mM KHCO_3_, 0.1 mM EDTA, pH 7.4) then washed with FACS buffer (2% newborn calf serum, 0.1% NaN_3_, 0.2 mM EDTA in PBS) ready for surface and intracellular staining.

### Flow Cytometry

Surface staining was performed in FACS buffer for 20 min at 4 °C including 10 μg/ml anti-mouse CD16/32 antibody (93, Biolegend) to block non-specific Fc-mediated antibody binding. The following antibodies were used: CD4 (RM4-5), CD44 (IM7), NKp46 (29A1.4), CD122 (TMβ1), NK1.1 (PK136), Thy1.2 (53-2.1), CD8α (53-6.7), TCRβ (H57-507), B220 (RA3-6B2), CD11b (MI/70), KLRG1 (2F1), CD80 (16-10A1), CD70 (FR70), H2kb (AF6-88.5), CD86 (GL-1), CD252 (RM134L) CD45 (30-F11) (Biolegend), Ly49G2 (4D11), CD8β (H35-17.2), TCRγδ (GL-3) (eBioscience), CD27 (LG.7F9), CD40 (3/23, BD), CD11c (HL3), IA/IE (M5/114.15.2) (BD Bioscience), IL15 (biotinylated, rabbit polyclonal, Peprotech) and IL15Rα (biotinylated, goat polyclonal, R&D). CD1d-tetramers were from NIH Tetramer Core Facility. Live/dead staining was performed using the LIVE/DEAD® Fixable Blue Dead Cell Stain Kit following manufacturer’s guidelines (Life Technologies). Intracellular staining was performed using the Cytofix/Cytoperm kit (BD Bioscience) and the following antibodies: Granzyme B (GB12, Caltag) and IFNγ (XMG1.2, eBioscience). Stained samples were measured on an LSRII flow cytometer (BD Bioscience) and analysed using FlowJo7 (TreeStar) or Flowlogic (Inivai Technologies) software. Cell populations were defined using living lymphocytes following exclusion of doublets using FSC and SSC. Lung lymphocytes were additionally defined as CD45^+^.

### ELISA

IL-15 and IL-15/IL-15Rα complex concentrations in mice sera, BMDC-supernatants and cell lysates were measured using specific ELISAs (R&D and eBioscience, respectively) according to manufacturer’s instructions.

### Tumor model

For lung metastasis model, age-matched female mice were used. The B16 murine melanoma cells were received from Jochen Huehn (Helmholtz Centre for Infection Research, Braunschweig, Germany) and cultured in RPMI-1640 medium supplemented with 10% FCS, 50 μg/ml Penicillin, 50 U/ml Streptomycin, 1 mM sodium pyruvate, 50 μM 2β-Mercaptoethanol, 25 mM HEPES. Cells were passaged once *in vivo* as follows: 2 × 10^e^5 cells were injected subcutaneously in C57BL/6J female mice, with tumors dissected on day 16. Melanoma tumor cells were recovered by digestion using Collagenase/Dispase (200 μg/ml, Roche) and DNASE I (20 μg/ml). Cells were cultured until confluent, aliquoted and stored in liquid nitrogen.

2 × 10^e^5 B16 melanoma cells in 200 μl of PBS were injected intravenously. At day 19, lungs were perfused by injection of pre-warmed PBS into the heart, dissected and the number of metastatic colonies in lungs were counted by a competent researcher blinded to the experimental design. Lung photographs were taken with a Canon 5D Mark 2 DSLR Camera and a Sigma MacroLens.

### Statistics

For group statistics comparison value, one way Anova with a Tukey’s multiple comparisons test has been used and a two way Anova with a Tukey’s multiple comparisons test has been used to compare *Il15*^−/−^, 71, 71-D-KO and D-KO strains, with significance depicted as *(p<=0.05), **(p<=0.01), ***(p<=0.001) and ns = non significant in all figures.

## Additional Information

**How to cite this article**: Polansky, J. K. *et al.* High dose CD11c-driven IL15 is sufficient to drive NK cell maturation and anti-tumor activity in a trans-presentation independent manner. *Sci. Rep.* **6**, 19699; doi: 10.1038/srep19699 (2016).

## Supplementary Material

Supplementary Information
