# Supplementary material for: High dose CD11c-driven IL15 is sufficient to drive NK cell maturation and anti-tumor activity in a trans-presentation independent manner
Source: Sci Rep. 2016 Jan 29;6:19699. doi: 10.1038/srep19699 (PMC4731790; doi:10.1038/srep19699)
Supplement: Supplementary Information [file srep19699-s1.pdf]

High dose CD11c-driven IL15 is sufficient to drive NK cell maturation and anti-tumor activity in a trans-presentation independent manner

Julia K. Polansky[1+*], Rajia Bahri[1,2*], Mylene Divivier[1], Erwin H. Duitman[1], Christina Vock[1], Diego A. Goyeneche-Patino[3], Zane Orinska[1*], Silvia Bulfone-Paus[2*]

[1]Research Center Borstel, 23845 Borstel, Germany; [2]Institute of Inflammation and Repair & MCCIR, University of Manchester, Manchester M13 9PT, UK, [3]Facultad de Medicina Veterinaria y Zootecnia, Universidad Cooperativa de Colombia, Bucaramanga, Colombia

[+]current address: Experimental Rheumatology, German Rheumatism Research Centre, Berlin, Germany
[*]These authors contributed equally to the work

Supplementary Materials & Methods

RNA extraction and IL15 expression analysis

Spleen and lung tissue samples (30mg) were homogenized in 600µl RLT buffer using the Omni TH2 homogenizer (Omni International). RNA extraction was performed using the RNeasy mini Kit (Qiagen). First strand cDNA was synthesized using 2µg of RNA, oligo-dT primer (5'-TTTTGTACAAGC(TTT)10-3') and the Superscript III reverse transcriptase (Life Technologies) according to the manufacturer's instructions.

IL15 gene expression in spleen and lung tissue samples was measured by quantitative RT-PCR using the LightCycler 480 (Roche Diagnostics), the ready-to-use hot start reaction mix (LightCycler® 480 probe master), the specific hydrolysis probes (Universal Probe Library, UPL) and primers, designed via the Universal Probe Library Assay Design Center (www.universalprobelibrary.com). IL15 expression values were normalized to the reference gene Hprt.

primer	primer Seq 5'-3'	UPL
mIL15_for	cagaggccaactggatagatg	#80
mIL15_rev	actgtcagtgtataaagtggtgtcaa	
Hprt_for	tcctcctcagaccgctttt	#95
Hprt_rev	cctggttcatcatcgctaatc	

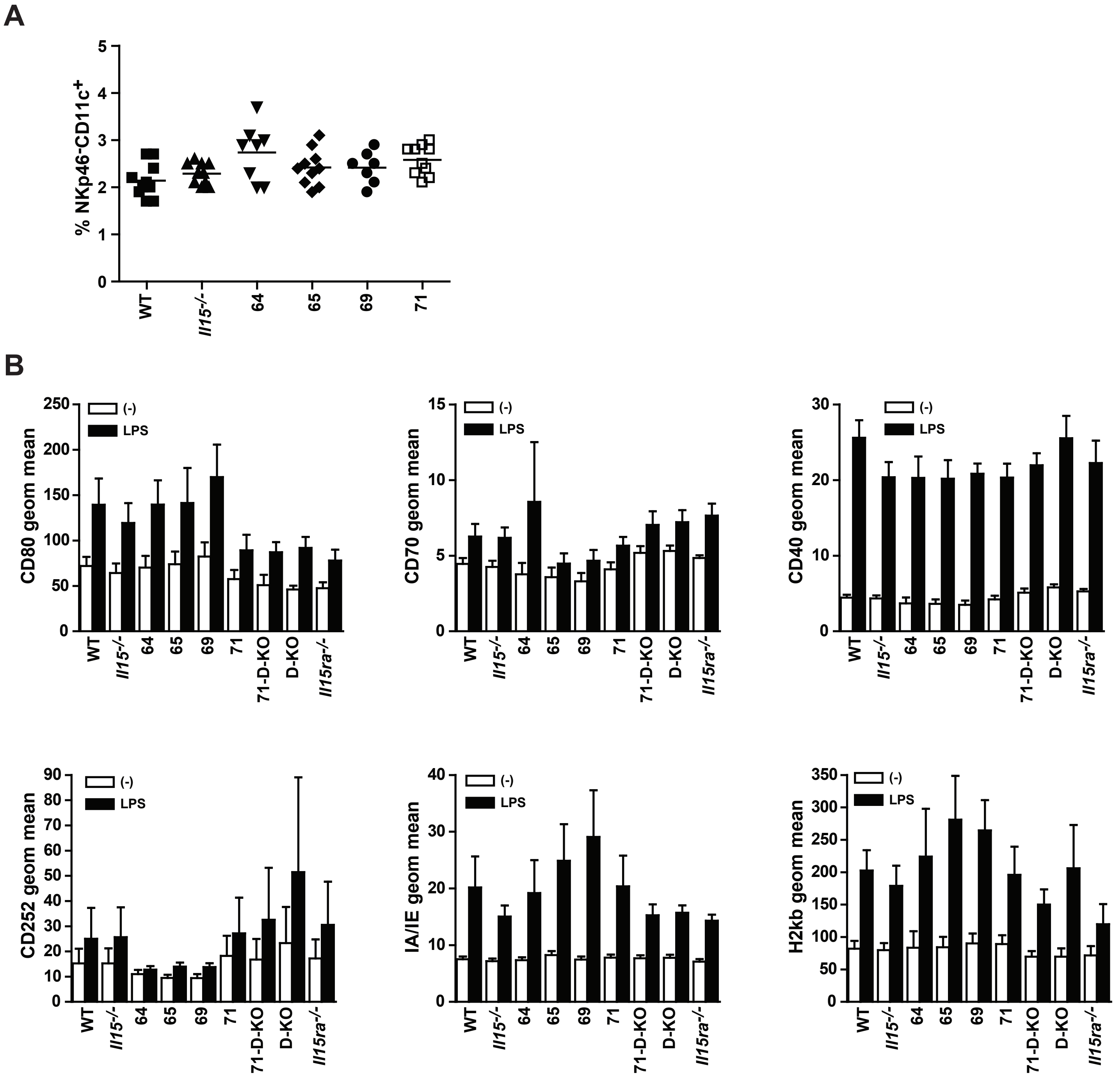

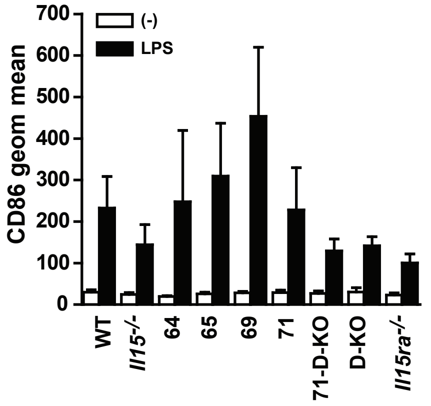

Suppl. Fig. S1: DC populations are unaltered in the transgenic mouse strains.

The frequency of CD11c+ DCs was analysed in spleen by flow cytometry and gated on CD11c+ NKp46- cell population (A). The phenotype of LPS-stimulated or untreated BMDCs generated from the different genotypes was analysed for the indicated surface makers by flow cytometry (B). No significant differences were observed between WT vs *Il15-/-* or transgenic strains and their controls (i.e. 64, 65, 69, 71 vs. *Il15-/-* and 71-D-KO vs. D-KO). Stimulated and unstimulated BMDCs were compared seperatly.

A

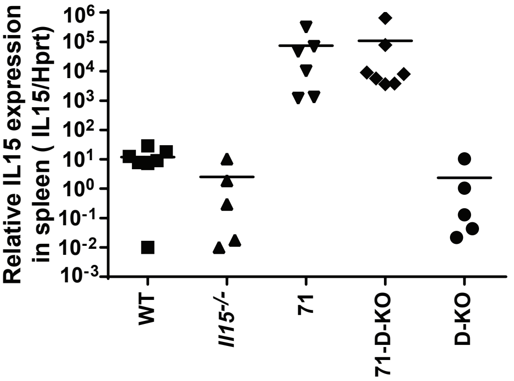

B

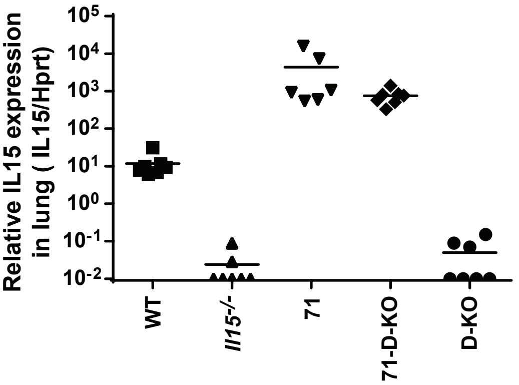

Suppl. Fig. S2: Level of IL15 mRNA in whole organ lysates measured by qRT-PCR.

IL15 gene expression in spleen (A) and lung (B) was measured by qRT-PCR in the different mouse strains (n=5-7). IL15 expression values were normalized to the reference gene Hprt. A and B summarize 2 independent experiments performed.
